# High Endothelial Venules and Other Blood Vessels: Critical Regulators of Lymphoid Organ Development and Function

**DOI:** 10.3389/fimmu.2017.00045

**Published:** 2017-02-03

**Authors:** Ann Ager

**Affiliations:** ^1^Division of Infection and Immunity, School of Medicine and Systems Immunity Research Institute, Cardiff University, Cardiff, UK

**Keywords:** blood vessels, high endothelial venules, peripheral node addressin, mucosal addressin, lymph nodes, tertiary lymphoid organs, ectopic lymphoid structures

## Abstract

The blood vasculature regulates both the development and function of secondary lymphoid organs by providing a portal for entry of hemopoietic cells. During the development of lymphoid organs in the embryo, blood vessels deliver lymphoid tissue inducer cells that initiate and sustain the development of lymphoid tissues. In adults, the blood vessels are structurally distinct from those in other organs due to the requirement for high levels of lymphocyte recruitment under non-inflammatory conditions. In lymph nodes (LNs) and Peyer’s patches, high endothelial venules (HEVs) especially adapted for lymphocyte trafficking form a spatially organized network of blood vessels, which controls both the type of lymphocyte and the site of entry into lymphoid tissues. Uniquely, HEVs express vascular addressins that regulate lymphocyte entry into lymphoid organs and are, therefore, critical to the function of lymphoid organs. Recent studies have demonstrated important roles for CD11c^+^ dendritic cells in the induction, as well as the maintenance, of vascular addressin expression and, therefore, the function of HEVs. Tertiary lymphoid organs (TLOs) are HEV containing LN-like structures that develop inside organized tissues undergoing chronic immune-mediated inflammation. In autoimmune lesions, the development of TLOs is thought to exacerbate disease. In cancerous tissues, the development of HEVs and TLOs is associated with improved patient outcomes in several cancers. Therefore, it is important to understand what drives the development of HEVs and TLOs and how these structures contribute to pathology. In several human diseases and experimental animal models of chronic inflammation, there are some similarities between the development and function of HEVs within LN and TLOs. This review will summarize current knowledge of how hemopoietic cells with lymphoid tissue-inducing, HEV-inducing, and HEV-maintaining properties are recruited from the bloodstream to induce the development and control the function of lymphoid organs.

## Introduction

Secondary lymphoid organs (SLOs) are sites in which immune responses are initiated and maintained in order to generate protective immunity against exogenous pathogens and tolerance to self-antigens and commensal organisms (1, 2). These specialized structures include lymph nodes (LNs), Peyer’s patches (PPs), tonsils, appendix, bronchus-associated lymphoid tissue (BALT), nasal-associated lymphoid tissue (NALT), isolated lymphoid follicles, and the spleen. All SLOs develop in the embryo, apart from NALT and BALT, which develop neonatally and in adults, respectively. As well as supplying oxygen and nutrients, the vasculature regulates the development of SLOs by recruiting a distinct population of hemopoietic cells that is essential to initiate lymphoid organogenesis. In adults, the blood vasculature in lymphoid organs is different from that found in other organs due to the requirement for efficient recruitment of lymphocytes under non-inflammatory, homeostatic conditions. Specialized blood vessels called high endothelial venules (HEVs) perform this function in all lymphoid organs except the spleen (3).

Tertiary lymphoid organs (TLOs) are lymph node-like immune cell clusters that develop inside non-lymphoid organs in response to chronic immune-mediated inflammation stimulated by persistent infections, chronic graft rejection, autoimmunity, and cancer (4). Defining features of TLOs are those that define SLOs: HEVs, lymphoid stromal cells, separate T-lymphocyte, and B-lymphocyte-rich compartments, follicular dendritic cell (FDC)-containing germinal centers, and antigen-presenting cells, including dendritic cells (DCs). However, in contrast to SLOs, TLOs are not encapsulated organs and are also known as tertiary lymphoid structures or ectopic lymphoid structures (ELS). TLOs are sites of active immune responses in autoimmune patients and animal models of autoimmunity (5, 6). There is evidence implicating TLOs in generating destructive immunity to self-antigens (7) although the relative contributions of TLOs and SLOs to disease progression are difficult to dissect during ongoing disease. Retrospective studies have correlated the presence of TLOs in resected solid cancers with prolonged patient outcome following resection of the primary cancer in several cancers (8). In some cancers, the density of HEVs alone predicted patient outcome (9, 10), indicating the critical role that HEVs play in orchestrating anti-cancer immunity. Importantly however, the formation of TLOs does not correlate with improved cancer patient outcome for all cancers. In virus-induced hepatic cellular carcinoma, TLOs promote carcinogenesis (11). In *Helicobacter pylori*-infected humans and mice, TLO development precedes carcinogenesis but whether TLOs promote the development of gastric cancer is not clear (12, 13). Therefore, there is much interest in how HEVs and TLOs develop and their exact roles in different chronic inflammatory diseases.

The development of LNs and PPs is well characterized, but how TLOs form is less clear because they develop during ongoing diseases. The similarities in structure between SLOs and TLOs suggest that underlying mechanisms driving their development may be conserved. Much attention has focused on the role of hemopoietic lymphoid tissue inducer (LTi) cells in driving the differentiation of local mesenchyme into lymphoid tissue organizer (LTo) cells during the development of LNs and PPs in mice (14–16). LTo cells are gp38^+^ fibroblasts and are precursors of lymphoid stromal cells such as fibroblast reticular cells (FRCs) and FDCs. Lymphotoxin (LT)-αβ on hemopoietic LTi cells engaging lymphotoxin β receptor (LTβR) on LTo cells plays a dominant role in differentiating LTo into chemokine-secreting, adhesion molecule expressing fibroblasts that are able to recruit and retain LTis in the developing lymphoid tissue. An equally important, but unanswered question, is how embryonic blood vessels deliver LTi cells from their site of generation in fetal liver to predetermined sites where LNs are to develop. A role for inflamed blood vessels in initiating TLO development is implied by the finding that myeloid cells recruited into inflamed tissues drive the differentiation of gp38^+^ fibroblasts that share properties with lymphoid stromal cells and are likely precursors of LTo cells (17). However, the mechanisms underlying myeloid cell recruitment by inflamed blood vessels were not determined in this study.

In adults, HEVs continually recruit naive and memory lymphocytes from the bloodstream into lymphoid organs in an antigen-independent manner where they survey DCs for cognate antigen (3). In peripheral LN draining the skin, HEVs express the peripheral addressin, peripheral node addressin (PNAd), whereas in PPs, HEVs express the mucosal addressin, mucosal addressin cell adhesion molecule (MAdCAM)-1. HEVs in some LNs (mesenteric, sacral, cervical) express both PNAd and MAdCAM-1. It has long been known that PNAd expression by HEV is actively maintained since PNAd levels on HEVs are rapidly downregulated following disruption of, or isolation from, the lymphoid microenvironment (18–20). Interestingly, HEVs in peripheral LN of newborn mice express the mucosal addressin but not the peripheral addressin (21, 22). During the first 2–3 weeks of life, HEV maturation is completed by a switch in vascular addressin expression from MAdCAM-1 to PNAd. How the vascular addressin switch is regulated and PNAd expression maintained on HEV in adult SLOs have been unclear. Recent studies have identified critical roles for CD11c^+^ DCs in the vascular addressin switch (23) as well as the maintenance of PNAd-expressing HEV in adult mice (24). Therefore, the blood vasculature plays a central role in recruiting distinct populations of hemopoietic cells at precise stages of lymphoid organ development that are essential to initiate lymphoid organogenesis, induce HEV maturation, and maintain fully differentiated HEV. These points will be addressed in greater detail in the following section.

A key step in the recruitment of lymphoid cells is their selection from the total pool of blood-borne leukocytes by binding to the inner blood vessel surface prior to transmigration across the vessel wall and entry into lymphoid organs. This involves a sequence of adhesive interactions between leukocytes and vascular endothelial (VE) cells, which can be divided into distinct stages of rolling and activation-induced arrest. In general, selectins mediate rolling and chemokines immobilized on the endothelial cell (EC) surface activate leukocyte integrins to arrest rolling cells. How hemopoietic cells with LT-inducing, HEV-inducing, and HEV-maintaining properties are recruited into lymphoid organs is central to understanding both the development of lymphoid tissues and the mechanisms regulating adaptive immune responses and disease pathologies.

## Blood Vessels and the Development of SLOs

**Figure 1:**
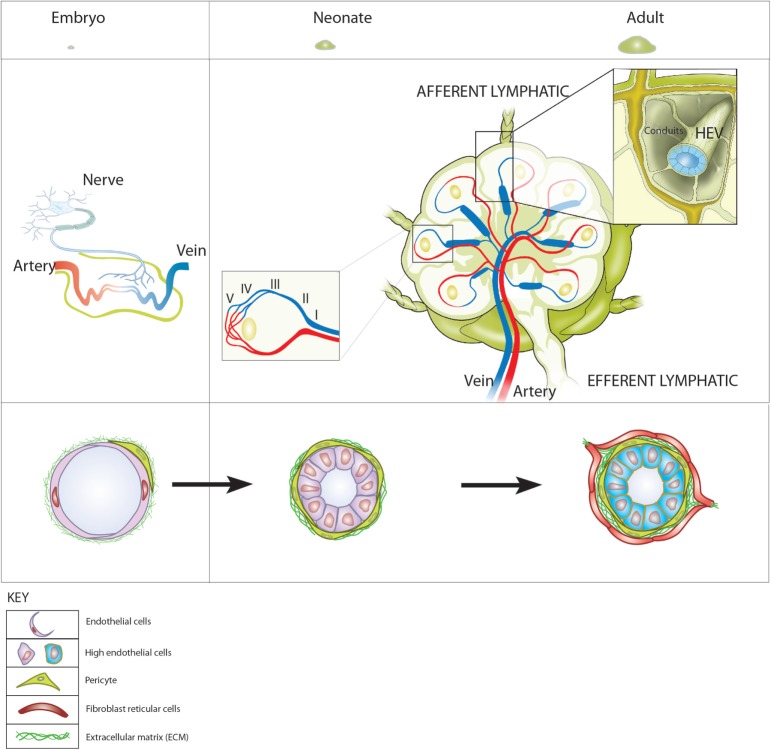
**Blood vessels and high endothelial venules (HEVs) in the development of lymph nodes (LNs)**. Top: LN development represented as relative sizes of (left) embryonic and (right) neonatal and adult LNs. Middle (left): in the embryo, neuronal stimulation induces retinoic acid-dependent expression of CXCL13 by mesenchymal cells that can be reverse transcytosed and presented on the inner surface of embryonic blood vessels. (Right) In LNs of adults, the HEV network extends from the cortical/paracortical junction adjacent to B cell follicles to large collecting veins in the hilar region gradually increasing in diameter from the smallest, order V venules to the largest order I venules (left hand inset). HEV connect directly to afferent lymphatics via fibroblast reticular cell-coated conduits that form the supporting internal scaffold on which lymphocytes and antigen presenting cells crawl during immunosurveillance (right hand insert). This enables the recruitment of fetal liver-derived CXCR5^+^ α4β7 integrin-expressing lymphoid tissue inducer cells to the sites where LN develop. (Right) At birth, structurally distinct MAdCAM-1-expressing HEV lined with cuboidal endothelial cells and supported by a thickened fibronectin containing basal lamina are visible. During the first weeks of life, MAdCAM-1-expressing HEV recruit CD11c^+^ neonatal migratory dendritic cells mobilized from the intestinal lamina propria, undergo a switch from MAdCAM-1 to peripheral node addressin expression and become ensheathed by fibroblast reticular cells. The vascular addressin switch is followed by rapid growth and a concomitant increase in cellularity and expansion in LN.

The earliest event in LN development is the formation of lymphatic vasculature around embryonic day 10.5 (E10.5) by budding from larger veins and establishing a primordial lymph sac or anlagen (Figure 1) (25). CD45^+^ CD4^+^ CD3^−^ IL7Rα^+^ RORγt^+^, Id2^+^ lymphoid tissue-inducing (LTi) cells (members of the group 3 category of innate lymphoid cells) are recruited into the anlagen and drive the development of gp38^+^ LTo cells from local mesenchyme (26). CXCL13 and IL7, produced by local mesenchymal fibroblasts in response to retinoic acid (RA) generated by neuronal stimulation, recruit CXCR5 and IL7 receptor-expressing LTi cells at sites where LNs are to develop (14). In the embryonic intestine, myeloid cell expression of the tyrosine kinase receptor, RET, is important to localize LTi to sites where PPs develop (27). LTαβ is upregulated on the surface of incoming LTis and stimulates LTβR-dependent upregulation of MAdCAM-1, vascular cell adhesion molecule (VCAM)-1, and intercellular adhesion molecule (ICAM)-1 and the secretion of homeostatic chemokines CXCL13, CCL19, and CCL21 by lymphoid stromal LTo cells. This enables LTo cells to retain recruited LTi cells in the developing anlagen. The continual recruitment and retention of LTi cells during embryogenesis is required to differentiate sufficient numbers of LTo cells to support the full development and organization of LNs and PPs (22).

During embryogenesis, LTi cells are generated in the fetal liver so how are they recruited to the sites at which LNs and PPs are to develop? A key finding is that all embryonic venous blood vessels express MAdCAM-1 (28). LTis express the integrin α4β7, which binds to MAdCAM-1 and α4β7-MAdCAM-1 binding is a dominant pathway for leukocyte recruitment from the bloodstream into developing LNs (21). In adults, homeostatic and inflammatory chemokines released by stromal cells are reverse transcytosed and presented on the inner blood vessel surface using Duffy antigen-related receptor-dependent and independent pathways (29–32). By analogy, CXCL13 released by activated fibroblasts presented on the inner surface of embryonic blood vessels together with cell surface MAdCAM-1, recruits CXCR5 expressing LTis from the bloodstream to initiate lymphoid organogenesis. Key questions, therefore, are what controls MAdCAM-1 expression on blood vessels in the embryo and is MAdCAM-1 essential for lymphoid organogenesis? MAdCAM-1 is expressed by all venous blood vessels as early as E9.5 in mice and expression is maintained throughout lymphoid organogenesis (28). In adults, MAdAM-1 expression by cultured EC is induced by classical Rel A/NF-κB signaling stimulated by factors such TNF-α, LT-α, IL-1-β, and LPS (33–35). Whether constitutive MAdCAM-1 expression on blood vessels in the LN anlagen is driven by NF-κB signaling or is ontologically determined, for example, by the transcription factor NKX2.3 (36), has not been determined. Interestingly, MAdCAM-1 expression by embryonic blood vessels is not essential for lymphoid organogenesis since LNs and PPs develop in MAdCAM-1 ko mice (37). LTis also express α4β1 integrin, which binds to VCAM-1 and could substitute for MAdCAM-1, however, mice globally deficient in VCAM-1 die in utero (38) and the role of VCAM-1 in lymphoid organogenesis is undetermined. ICAM-1 expression by LTo cells is not essential for lymphoid organogenesis since LNs develop in ICAM-1-deficient mice (39). However, MAdCAM-1, VCAM-1, and ICAM-1 are not redundant in adult mice because they regulate lymphocyte recirculation through SLOs and/or recruitment to inflamed tissues (1, 3).

Lymph nodes and PPs do not develop in LTα ko mice (40). Administration of an LTβR agonist to pregnant mice bypasses the requirement for LTi cells and induces LN development (41). The initiation of LN and PP organogenesis is time restricted; mesenteric LN starts around E9–10 and PPs around E16 with peripheral LN (axillary, brachial, inguinal, and popliteal) between E10 and E16. However, exogenous administration of LTβR agonists outside of these times will not initiate LN formation. Administration of antagonistic LTβR-Ig to pregnant wildtype mice also blocks the development of LN in a similar time-dependent manner (42). What controls the timing of LN development is not clear but one possibility is the ability of embryonic blood vessels to recruit LTi cells. For example, the expression of sufficient MAdCAM-1, immobilized chemokines, or other, as yet unidentified, homing-associated molecules that recruit LTi cells from the bloodstream into to the LN anlagen may be time dependent. The local generation of RA drives lymphoid organogenesis and RA is required to mature developing blood vessels in the embryo (43). It is possible that locally generated RA facilitates LTi recruitment by promoting the expression or activity of homing-associated molecules on growing vessels in the LN anlagen, but we know very little about when RA is produced and how this is regulated.

Although LTαβ–LTβR is a dominant NF-κB signaling pathway and CXCL13 a dominant chemokine in the development of peripheral LN and PP, other pathways can substitute during the development of mucosal and visceral draining LN. For example, mesenteric, cervical (submandibular), sacral, and lumbar LN (44) develop in LTβ-, LTβR-, or CXCL13-deficient mice (15, 22). LTβR engagement by membrane LTαβ stimulates both classical NF-κB signaling *via* Iκκβ and p50/RelA as well as non-classical signaling *via* Iκκα and p52/RelB, and there is considerable interplay between these two pathways (45). LN and PP do not develop in mice globally deficient in either RelA or Rel B, key components of classical and non-classical NF-κB signaling, respectively, although the impact of classical NF-κB signaling on LN development may be *via* upregulation of non-classical NF-κB signaling substrates such as NF-κB2 and RelB (46, 47). In mice deficient in NFkB2, the substrate for p52 in the non-classical pathway, mesenteric and some peripheral LN develop but lymphoid organs that form later in embryogenesis (inguinal, popliteal, and PPs) are small or do not develop at all (48). It is suggested that p50/RelB can substitute for p52/RelB in these mice but the signal strength is weaker and so, although LTi cells are recruited, the induction of CAMs is not enough to retain sufficient LTi cells to maintain development and organize the full structure of late developing lymphoid organs. Non-classical NF-κB signaling is important for the proper development of HEVs since the small LN that develop in LTβ- or NFkB2-deficient mice have poorly developed HEVs, as do peripheral LNs that develop in mice expressing a signaling-deficient mutant of the non-classical NK-κB pathway, Iκκα (48, 49). The widespread expression of LTβR on the developing vasculature as well as LTo cells makes it difficult to assess their relative contributions to the development of lymphoid organs or of HEVs in mice globally deficient in either LTαβ–LTβR or following administration of antagonistic LTβR-Ig. Recent studies have shown that the development of peripheral LNs in 25–40% of pups is completely blocked in mice selectively deficient in LTβR in blood and lymphatic ECs, but the underlying mechanism is not clear (50). Further work will be required to dissect the roles of NF-κB signaling in the blood vs the lymphatic vasculature during lymphoid organogenesis and how this integrates into the scheme of lymphoid organogenesis driven by gp38^+^ lymphoid stromal LTo cells.

## The Development and Function of High Endothelial Venules in SLOs

### The Vascular Addressin Switch

The recruitment of naïve T and B cells into all lymphoid organs, apart from spleen, is dependent on the differentiation of a subset of blood vessels into HEVs. Structurally distinct HEVs are not apparent in LN of mice until birth when a branching network of HEV blood vessels starts to organize around B cell follicles during the first days after birth in PPs (28). A key event in neonatal maturation and expansion of LN is a switch in vascular addressin expression by HEV (21). In LNs of newborn mice, all HEVs express the mucosal addressin MAdCAM-1; during the first few weeks of life MAdCAM-1 is downregulated and expression of the PNAd is upregulated. PNAd comprises a mixture of ligands for L-selectin (CD34, podocalyxin, GlyCAM-1, MAdCAM-1, nepmucin, and endomucin) that are modified by 6-sulfo sialyl Lewis^x^ on extended core 1 O-linked oligosaccharides and detected by monoclonal antibody MECA79 (51). PNAd expressed on the inner, apical surface of HEVs co-operates with the arrest chemokine CCL21 to select L-selectin/CD62L^+^, CCR7^+^ lymphocytes from the bloodstream for entry into LN allowing postnatal colonization of LN by naive T and B lymphocytes as they are released into the circulation from the thymus and fetal liver/bone marrow, respectively. Interestingly, PNAd is also expressed on the basolateral surfaces of ECs lining HEVs but it is not involved in recruiting lymphocytes directly from the bloodstream. Distinct sulfotransferases generate apical and basolateral PNAd and the expression of GlcNAc6ST-2 (HEC-6ST; CHST4), which is required to generate apically expessed PNAd, is restricted to HEV ECs, whereas GlcNAc6ST-1 (CHST2), which generates basolaterally expressed PNAd, is more widely expressed in stromal cells (51). The PNAd-expressing HEV network grows alongside the growth of other stromal components resulting in a rapid increase in the size and cellularity of LN during the first weeks of life (23). Although PNAd is a well-characterized ligand for L-selectin, it only accounts for 50% of L-selectin-dependent recruitment into peripheral LNs. Important non-PNAd ligands on HEV that regulate L-selectin-dependent recruitment into peripheral LN include 6-sulpho sialyl Lewis X modified N-linked glycans such as CD34, as well as a minor role for core 2-branched O-linked glycans (51, 52).

Vascular addressins expressed on the inner, luminal surface of ECs lining HEV bind to homing receptors on lymphocytes; therefore, addressins define the specialized property of HEV in driving lymphocyte recruitment into LNs and PPs. PNAd was originally described in HEVs of peripheral LNs draining the skin (inguinal, axillary, brachial, and popliteal) (53) and MAdCAM-1 in mucosal-associated lymphoid tissues such as PPs (54). However, analysis of other mucosal and viscera-draining SLOs in mice shows that PNAd expression is upregulated and MAdCAM-1 variably downregulated. For example, MAdCAM-1 expression is not completely downregulated in mesenteric and sacral LNs where individual high endothelial cells (HECs) co-express PNAd and MAdCAM-1. However, mucosal addressin is completely downregulated in lymphoid tissues draining other mucosal sites such as cervical (submandibular) LN or NALT (4). Similarly, LNs draining visceral organs, such as lumbar LN, express PNAd and not mucosal addressin (44); therefore PNAd expression is not restricted to subcutaneous LN and is widely expressed by mucosal and visceral LN in mice. PNAd expression was originally reported to be restricted to the basolateral surfaces of HEVs in PPs (54). However, in different mouse strains and other species, PNAd expression in PPs is comparable with that in peripheral LN, which may reflect increased antigenic stimulation (51). Why MAdCAM-1 expression is maintained by HEVs in PPs and some mucosal LN whereas other HEVs completely switch to PNAd expression is not clear, but MAdCAM-1 expression is regulated by the transcription factor NKX2.3 (55).

### The Connection with Afferent Lymph

A unique feature of HEV is the connection with afferent lymph. HEVs are supported by a perivascular sheath composed of FRCs that are connected to the FRC coated conduit system within LN. The conduit system allows communication between afferent lymph and HEV whereby incoming lymph-borne soluble factors, such as chemokines and cytokines, are delivered directly to the basal lamina of HEV (Figure 1) (56). Early studies showed that ligation of afferent lymphatics, which drain fluid and immune cells into popliteal LN, resulted in a gradual flattening of HECs and although some PNAd expression was retained it was restricted to the basolateral surfaces of HECs (18, 19, 57). Other changes in HECs included a transient induction of MAdCAM-1 expression, which peaked 4 days following deafferentization suggesting reversion of mature HEVs to an immature state. Lymphocyte recruitment by HEV decreased and was no longer detectable 8 days after deafferentization. HEVs reverted to fully functional vessels expressing PNAd at the luminal surface following regrowth of afferent lymphatics demonstrating the complete reversibility of HEV differentiation. Early studies indicated a dominant role for LTβR signaling in maintaining PNAd expression since chronic administration of LTβR blocking reagents to adult mice recapitulates the effects of deafferentization in that HEVs downregulate PNAd and MAdCAM-1, and therefore lose the ability to support lymphocyte homing (58). The flattened appearance of PNAd-positive HEV in deafferentized LNs is seen in peripheral LNs that develop in mice in the absence of endothelial expression of LTβR (50) and in inguinal LNs developing in the absence of either NFKB2 or IKKα (48, 49), indicating a key role for LTβR stimulated non-canonical NFκB signaling in driving luminal expression of PNAd by HECs. Which cells engage LTβR on HECs to induce and maintain PNAd expression has been unclear, however, recent studies identify dominant roles for CD11c^+^ DCs in the induction, as well as the maintenance, of PNAd expression by HEV.

### Role of CD11c^+^ Cells in the Vascular Addressin Switch

An important finding is that during the first weeks of life CD11c^+^, CD11b^+^, CD103^+^ DCs mobilized from the gut lamina propria in response to microbial colonization induce the switch from mucosal to peripheral addressin expression by HEV in peripheral LN (23). RA-dependent signaling is critical for these so-called neonatal migratory DCs to induce the vascular switch; cells that do not express retinaldeyhde dehydrogenase (RALDH), which generates RA from retinaldehyde, a derivative of Vitamin A, are unable to induce the vascular addressin switch. This important finding came from studies of adult germ-free mice in which peripheral LN HEVs express MAdCAM-1, but not PNAd, and LN are small without clear compartmentalization into T and B-cell areas, similar to LN in newborn mice. When germ-free mice were co-housed with conventionally housed mice, HEVs underwent the vascular addressin switch with consequent increases in lymphocyte homing and LN cellularity. These effects could be reproduced by administration of RALDH^+^ neonatal migratory DCs isolated from conventionally housed adult mice to young germ-free mice. Migration of neonatal migratory DCs to peripheral LN was dependent on MAdCAM-1 expression by HEVs in peripheral LN. Interestingly, the migration of these cells to peripheral LN was highest around 2 weeks after birth and subsided after 6 weeks, presumably because of the loss of MAdCAM-1 expression by peripheral LN HEVs.

In adult mice, CD11c^+^ cells have been shown to maintain PNAd-expressing, fully functional HEVs by stimulating LTβR-dependent signaling in HECs (24, 50). Moussion and Girard used transgenic mice expressing the diphtheria toxin (DT) receptor under the CD11c promoter (CD11c-DTR mice) and DT to deplete CD11c^+^ cells (24). They noted a gradual loss of PNAd expression, a transient expression of MAdCAM-1, and a reduction in lymphocyte entry *via* HEVs over 8 days, which was prevented by administration of exogenously generated LTαβ-expressing CD11c^+^ DCs. Since ligands for LTβR are membrane bound, LTαβ DCs might be expected to make frequent contacts with HECs under homeostatic conditions in order to maintain PNAd expression. Migration of DCs into LN of adult mice has focused on entry from surrounding tissues *via* incoming, afferent lymphatics. DCs entering *via* this route do not come into cellular contact with HECs since they do not penetrate the surrounding pericytic sheath (59, 60). However, DCs entering from surrounding tissues *via* afferent lymphatics do locate around HEVs (61); it is therefore possible that protruding DC membranes or exosomes released by DC (62, 63) could cross the pericytic sheath and contact with HECs. Precursors of classical DCs that are recruited from the bloodstream into LN of adult mice spend up to 5 h in the walls of HEVs are a possible HEV-maintaining cell. In this scenario, the critical role of afferent lymph in maintaining fully differentiated HEVs is not as an entry point for DCs but to supply the chemoattractants necessary to recruit LT-expressing DCs from the bloodstream, enabling DC-HEC contact to sustain LTβR signaling.

Interestingly, CCR7^+^ expression by CD11c^+^ cells is required to control the homeostatic expansion of PNAd-expessing HEV but in the absence of CCR7 expession, CD11c^+^ cells are still able to induce the switch in addressin expression to PNAd (64). The increase in size of the HEV network and associated increase in LN cellularity and volume is due, in part, to the release of angiogenic stimuli such as VEGF-A by FRC and DCs (65, 66). These findings suggest that the differentiation of fully functional, PNAd-expressing HEV and the expansion of the HEV network are controlled by different types of CD11c^+^ cells in adult mice. It will be interesting to determine whether RA signaling is required for CD11c^+^ DCs to maintain PNAd expression and expand HEV in adult mice as has been shown in neonates (23).

### High Endothelial Venules in Homeostatic Lymphocyte Trafficking

Together, the SLOs are uniquely placed to survey the whole body for incoming pathogens and self-antigens derived from the skin, mucosal surfaces, or directly into the bloodstream, mount an appropriate immune response and clear invading pathogens from the body. To achieve this, rare lymphocytes with appropriate antigen receptor must be selected from the total pool of lymphocytes each with its own unique receptor; this occurs inside SLOs. Pathogens are first degraded and presented as MHC-binding peptides on DCs bound to gp38^+^ FRCs. The total pool of lymphocytes in the body is then exposed to pathogen peptide-enriched DCs. Lymphocytes with antigen receptors able to respond to the level of peptide presented are removed from the circulating pool of cells and undergo proliferation and differentiation to effector, memory, or regulatory lymphocytes before returning to the circulation. Blood vessels are critical components of adaptive immunity since they recruit naïve and central memory lymphocyte irrespective of antigen receptor specificity and deliver them to DCs inside lymphoid organ under homeostatic conditions. Postcapillary venules (PCV) lined with continuous ECs are the preferred sites of lymphocyte extravasation in LNs and other organs such as the skin and GI tract. The increase in diameter as blood vessels transition from capillaries to PCV alters hemodynamics such that leukocytes move to the outer stream or margin of flowing blood adjacent to the inner surface of the vessel (67). Here lymphocytes tether, roll, and arrest on the inside surface without obstructing flow that would happen in smaller capillaries. In other organs where the microvessels are lined with sinusoidal endothelium, such as the spleen, liver, and bone marrow, shear stress at the vessel wall–blood interface may be significantly reduced that the requirement for selectins for capture from flowing blood is not necessary and other homing-associated molecules such as leukocyte integrins perform this role (67). In LNs and PPs, the trafficking of naïve lymphocytes is restricted to specialized postcapillary venules called HEVs. These vessels are structurally adapted to support large-scale lymphocyte trafficking without compromising vascular integrity. The spleen does not have HEV and, in mice, lymphocytes enter the spleen from capillaries in the marginal zone using incompletely defined molecular recognition pathways. The structure of human spleen differs from that in mice and the route of lymphocyte entry has not been identified (68).

Arteries feeding the LN enter at the hilar region and arborize into nutrient- and oxygen-transporting capillary beds surrounding B cell follicles in the outer cortex and in the T cell zone. The capillary beds lead directly (or indirectly *via* arteriovenous shunts) into the postcapillary venular network, which extends throughout the paracortex (T cell area) of the LN; HEVs form part of this postcapillary network (Figure 1). HEVs are readily distinguished from other blood vessels; the cuboidal (high) ECs, which line HEV and give these vessels their name, contrast with flat ECs lining other vessels. In addition, HEVs are supported by a thickened basal lamina comprising overlapping pericytes and a perivascular sheath of FRC that together generate a structural and functional unit exquisitely adapted to support high levels of lymphocyte recruitment and transendothelial migration to deliver lymphocytes to the LN parenchyma. The ECs lining HEVs express a number of pan-endothelial markers such as VE-cadherin and CD31 as well as the master venous regulator Nr2f2 (69), which suggests that they differentiate from local postcapillary venules. Invitavital imaging of lymphocyte recruitment allows HEVs to be ordered according to size and location. The smallest vessels (order V) are found at the junction between the B cell-enriched cortex and the T cell-enriched paracortex. HEVs gradually increase in size to larger vessels (order II) at the junction between the paracortex and medulla where they merge into order I collecting venules which drain into hilar vein (70) (Figure 1). The majority of lymphocytes recruited into LN and PPs are from the higher order vessels III–V. In peripheral LN, L-selectin dependent recruitment from lower order vessels does occur but the vascular ligand on HEC is distinct from PNAd (71). How this complex branching network of differentiated HEVs develops and is maintained in LN is not clear but it is likely to be connected with development of the highly organized secondary structures of FRC-coated conduits that connect the draining lymphatics with the HEVs (29). The dominant roles of LTβR signaling in maintaining fully differentiated PNAd-expressing HEVs as well as T/B compartmentalization in LNs are likely to cooperate in forming an organized HEV network.

The ability to image the behavior of leukocytes inside postcapillary venules using intravital microscopy has identified the molecular interactions between lymphocytes and HEVs in LN and PPs that control recruitment from flowing blood. The multistep adhesion cascade describes the sequence of tethering, rolling and activation-induced arrest, which selects lymphocytes for transendothelial migration and entry into lymphoid organs. The cascade is best exemplified by the fact that neutrophils undergo L-selectin-depending rolling in HEVs but they are unable to undergo activation-induced arrest by CCL21 immobilized on the inner HEV surface because they do not express CCR7 and are, therefore, not recruited into LN under homeostatic conditions (70). The selectin, chemokines, and integrins that regulate rolling and activation-induced arrest of naive and central memory T and B cells and the non-random recruitment of T and B lymphocytes in HEV of LN and PP of mice have been described in detail elsewhere (1, 3, 72–74). The regulated expression of peripheral node and mucosal addressins by HEV and their roles in lymphocyte recruitment are conserved in LNs of larger animals as well as humans indicating that studies in mice have clinical relevance (75–79). This review will summarize recent advances in understanding the structure and function of HEV and its role in regulating adaptive immunity.

In LNs, L-selectin mediates tethering and rolling of lymphocytes. In comparison with T cells, B cells express lower L-selectin and are reduced in LNs but highly enriched in PPs. Although L-selectin supports lymphocyte rolling in HEVs of PPs, it is not a dominant homing molecule under homeostatic conditions since, in contrast to peripheral LN, the cellularity of PPs in L-selectin deficient mice is not reduced (80). The B-cell expressed lectin that supports preferential recruitment by HEVs of PPs was unknown until a detailed comparison of transcriptomes expressed by HECs isolated from PPs and peripheral LN of mice demonstrated preferential expression of the enzyme β-galactoside α-2,6-sialyltransferease I (ST6GaI 1) by PP HECs. ST6Gal 1 generates high affinity α-2,6-sialylated glycan ligands for the B cell lectin CD22 (Siglec-2) and was shown to function as a B cell selective mucosal addressin in PPs (69).

Apart from addressin expression and their location inside LN, a characteristic histological feature of HEV is lymphocytes embedded in the walls of HEV, which suggests that transmigration is a rate-determining step in lymphocyte recruitment from the bloodstream into the LN parenchyma. Transmigration is a rapid event taking 3 min to cross the endothelial lining and 10 min to complete migration across the underlying basal lamina (81–83). The molecules and signaling pathways that regulate transmigration are not completely understood. Whether lymphocytes move through the junctions between HECs (paracellular route) or penetrate the EC cytoplasm (transcellular route) has long been debated (84, 85). It is important to understand how lymphocytes transmigrate the walls of HEV since the potential for bi-directional signaling in lymphocytes and HECs may prime transmigrating lymphocytes for interstitial motility and immunosurveillance. Recent studies have demonstrated that lymphocytes are held in the so-called HEV pockets that are extracellular spaces between HECs and the surrounding pericyte-containing basal lamina (86). The accumulation of transmigrating lymphocytes, particularly T cells, contributes to the height of HECs (81, 87). Residence in HEV pockets provides an opportunity for cellular contacts with HECs or with other transmigrating cells, such as DCs, which may facilitate rapid transmigration in comparison with vessels lined with flat EC (81). It is also possible that antigen could be passed from the basolateral adjoining conduits to DCs inside HEV pockets for presentation to incoming lymphocytes. HEVs are contractile vessels responding to locally released vasoactive agents (88). Arterial pressure and vasoconstriction can regulate the height of HECs measured in histological sections of LN (89). Dynamic changes in shear stress at the vessel wall interface generated by HEV contractility may enhance the capture of lymphocytes from flowing blood.

Lymphocytes form very close, intercellular, gap-like junctions of 2–4 nm with HECs during transmigration (90), which may be the *in vivo* equivalent of the docking structures between lymphocytes and ECs reported *in vitro* (91, 92). ECs lining HEVs express a range of junctional proteins that are found in other vascular beds, including VE-cadherin, CD31, JAM-A, JAM-B, JAM-C, and ESAM-1 (93). Engagement of EC junctional proteins by complementary receptors on lymphocytes may regulate migration across the endothelial lining of HEVs, as shown for leukocytes transmigrating inflamed blood vessels (67). Unlike other types of endothelium, HEV ECs lack tight junctions and vascular specific claudin-5. These distinct structural features may facilitate paracellular transmigration of lymphocytes and/or lymphocyte accumulation inside HEV pockets.

Some progress has been made in dissecting the molecular events driving entry and exit of lymphocytes from HEV pockets and subsequent penetration of the underlying basal lamina to enter the LN parenchyma (94). Lymphocytes are retained in HEV pockets of FTY720 treated mice, and this was thought to be due to lack of space in the LN parenchyma (86). However, as well as blocking lymphocyte exit from LN *via* lymphatics, FTY720 inhibits T cell recruitment from the bloodstream across HEVs (95). An alternative explanation is that incoming T cells drive the exit from HEV pockets and, in the absence of incoming T cells in FTY720 treated mice, lymphocytes do not complete transmigration, as has been shown *in vitro* (96). There are other mechanisms controlling lymphocyte retention in HEV pockets. T cells accumulate in the endothelial lining of HEVs in mice treated with dual specificity MMP/ADAM inhibitors (87), suggesting that exit from HEV pockets may be metalloproteinase-dependent. L-selectin is proteolytically cleaved from T cells as they transmigrate HEVs (97) and lymphocytes expressing a metalloproteinase-resistant mutant of L-selectin take longer to transmigrate the endothelial lining of HEVs (98). Proteolytic shedding of L-selectin following cross-linking by basolaterally expressed ligands such as PNAd may polarize transmigrating T cells, as recently demonstrated in monocytes (99) and neutrophils (100) and allow directed migration into the LN parenchyma in response to HEC-derived lipids such as autoaxin and lymphoid stromal cell-derived chemokines such as CCL19 and CCL21 (101, 102).

### The Impact of Immunization and Infection on High Endothelial Venules

There are marked changes to HEVs in LNs draining sites of immunization or infection. These include increased blood flow, expansion of the HEV network, and marked changes in expression of homing-associated molecules. Changes in HEV function cooperate to alter the size and composition of the leukocyte infiltrate. In particular, altered expression of homing molecules on HEV enables the recruitment of activated lymphocytes and innate immune cells that are normally excluded by HEVs because they lack expression of L-selectin or CCR7. The impact of altered immune cell recruitment *via* HEV on ongoing immunity is only just starting to be analyzed (94).

Innate immune cells remodel feeding arterioles to increase blood supply and, thereby, increase the delivery of naïve lymphocytes into draining LNs (103). This allows a major fraction of the full repertoire of lymphocytes to pass through antigen-activated LNs within a few days (104). The HEV network grows to accommodate the increase in blood supply and the accompanying increase in lymphocyte trafficking. For example, the total length of HEVs increases threefold from approximately 10–30 cm in antigen-reactive LN in parallel with a threefold size increase of LN volume (105). LT and VEGF family members regulate expansion of the HEV network. LTβR activation of FRCs by DCs induces release of VEGF-A, an angiogenic factor that stimulates HEV growth (65, 106). VEGF-A is also produced by activated DCs, which, following injection into the skin, stimulate the growth of HEV in LN draining the site of injection (66).

Other changes to HEV in antigen-challenged LN relate directly to links with afferent lymphatics. For example, the plasticity of HEV revealed by afferent lymphatic ligation (19) or isolation from the LN environment (20) is directly relevant to adaptive immune responses. Antigen administration is accompanied by a temporary shutdown of afferent lymphatics between days 2 and 5 and concomitant dedifferentiation of HEV (107). Interestingly, the loss of luminal PNAd by HEV following antigen administration is accompanied by transient induction of MAdCAM-1 as found following deafferentization or CD11c^+^ cell depletion. By limiting the influx of lymphocytes, the loss of PNAd expression may serve to prevent the dilution of lymphocytes already in contact with antigen-laden DCs and promote the generation of memory cells. The reversion of PNAd-expressing HEV to MAdCAM-1-expressing HEV may allow entry of LTis or other α4β7 integrin-expressing innate lymphoid cells (108), for repair or remodeling of LN during the later stages of virus infection to restore preinfection architecture (109).

Vascular changes in antigen-stimulated LNs are accompanied by increased growth of afferent lymphatics and remodeling of the FRC network that alters the availability of chemokines for presentation by HEVs. For example, the dramatic reduction in CCL21 and CXCL13 expression by LN stroma following viral or bacterial infection (110) impacts on T and B lymphocyte recruitment since stromal cell-derived chemokines are presented by HEVs to blood-borne lymphocytes (32). The increased delivery of DCs and other immune cells mobilized from inflamed tissues *via* expanded afferent lymphatics may have, as yet unexplored, effects on HEV function. However, as the immune response subsides, CD11c^+^ cells are required to remodel the perivascular FRC sheath surrounding HEVs and restore vascular permeability and HEV function (111). These observations suggest a regulatory loop based on HEV that controls the size of LN, preventing uncontrolled growth of inflamed lymphoid tissue. How the structure of LNs and other secondary lymphoid tissues is restored to the preactivated state is largely unknown. This is likely to be relevant to understanding TLOs that develop in chronically inflamed tissues.

## Blood Vessels, High Endothelial Venules, and the Development of TLOs

Tertiary lymphoid organs or ELS develop inside non-lymphoid organs. TLOs form in response to chronic immune-mediated inflammation stimulated by persistent antigens such as infection, allograft rejection or ulcerative colitis but also in several autoimmune conditions such as rheumatoid arthritis and Hashimoto’s thyroiditis (112). TLOs have been reported by histology in biopsied or surgically removed clinical tissues from the majority of organs in the body including the CNS and atherosclerotic aorta. TLOs are also found associated with cancerous tissues. TLOs are highly organized lymph node-like structures containing discrete T and B-cell rich areas supported by stromal cells that share markers with FRCs and FDCs in LNs. TLOs contain PNAd-expressing blood vessels that resemble structurally distinct HEV in LNs; they are the presumed sites of entry of blood-borne lymphocyte and, therefore, critical to the function of TLOs. PNAd-expressing blood vessels lined by flat ECs are found inside cancer-induced ectopic lymphoid aggregates that are not organized into distinct T/B cell areas. These could represent immature HEV-containing structures in the process of forming TLOs, or TLOs that are distintegrating during the resolution of chronic inflammation (113). Interestingly, PNAd-expressing blood vessels that form following depletion of Foxp3^+^ regulatory T cells from cancer-bearing mice are not associated with histologically distinct, lymphoid cell aggregates (114) which indicates that HEV neogenesis can precede lymphoid neo-organogenesis. The development of HEV in the absence of full-blown TLOs correlates with cancer regression in this experimental model highlighting the important role of HEV in controlling immunity to cancers. Interestingly gp38^+^ (podoplanin) stromal cells are induced locally by CD11b^+^ myeloid cells recruited to inflamed skin of mice, recapitulating the earliest stages of lymphoid stromal cell development, but whether CD11b^+^ myeloid cells drive the development HEV and/or TLOs in chronic inflammation is not known (17).

How HEVs form during ongoing chronic diseases is difficult to dissect but insights to the stimuli and signaling pathways that control HEV neogenesis and function have come from experimental studies in mice. In experimental animals, organized lymphocytic infiltrates containing PNAd-expressing blood vessels develop in exocrine tissue of the pancreas and thyroid in response to ectopic expression of homeostatic chemokines and cytokines that control LN development (115–118) and virus-induced autoantibody production in the salivary gland (119). However, as found in chronic diseases, the size, location, and composition of lymphoid infiltrates that develop at ectopic sites vary depending on the stimulus. For example, when LTα is expressed in pancreatic β cells PNAd expressing blood vessels are found inside the small lymphoid infiltrates that form around some islets (120). Development is independent of endogenous LTβ and dependent on signaling *via* the type I TNFR. However, the infiltrates comprise mainly memory T cells that express low levels of L-selectin, which may be due to the predominantly abluminal expression of PNAd, which is unable to recruit L-selectin expressing lymphocytes (118). Co-expression of both LTα and LTβ and consequent LTβR signaling in the exocrine pancreas is required to develop large, organized lymphoid aggregates that contain HEV expressing PNAd at the luminal surface and, as in LNs, these ectopic TLOs are highly enriched in L-selectin-expressing T and B lymphocytes. LTαβ expressing cells other than LTis drive TLO formation, such as T and B cells which upregulate LTαβ in response to ectopic expression of CCL21 and CXCL13, respectively (116). PNAd expressing, structurally distinct HEV develop within 5 days of transferring T cells to RAG-deficient mice expressing CCL21 under the thyroglubulin promoter and, as found in LN, HEV development is dependent on LTαβ–LTβR signaling and DCs (117, 121). As in LN, the development of PNAd expressing HEV in cytokine or chemokine-induced TLOs is stunted in mice deficient in either LTα or LTβ. PNAd-expressing blood vessels lack the HEV-restricted sulfotransferase and, therefore, luminal expression of PNAd, and are lined with flat ECs typical of immature HEVs in LN unable to support high levels of lymphocyte traffic. The lack of HEV maturation in the absence of LTα or LTβ correlates with and, most likely, contributes to the reduced size and cellularity of lymphoid infiltrates in these mice (118).

The development of PNAd-expressing HEV in mouse models of cytokine- or inflammation-induced cancer correlates with increased T cell infiltration and priming and reduced tumor growth (114, 122, 123). In tumor cell transplant models, PNAd expression is induced on tumor blood vessels by infiltrating tumor-specific effector CD8^+^ T cells as well as NK cells (124). In marked contrast to HEV development in LN and TLO, PNAd expression is not dependent on LTβR signaling but is stimulated by CD8^+^ T and NK cell-derived LTα3 activation of TNFR. Interestingly, expression of the arrest chemokine CCL21 is not induced by the same stimuli that induce PNAd expression, but instead by IFN-γ released by activated T and NK cells. However, the tumor-associated HEVs are distinct from conventional mature HEVs in LN since PNAd expression is exceptionally low and the endothelial lining is flat, rather than the characteristic, LTβR-dependent cuboidal morphology found in LNs (50). Although comprising <10% of the tumor vascular network and lined by flat EC, these PNAd-expressing tumor blood vessels are functional in that they recruit naïve, L-selectin-expressing T cells from the bloodstream into the tumor where they are activated to kill tumor tissue (124). These findings suggest that TNFR signaling in ECs stimulates the development of PNAd-expressing blood vessels resembling immature HEVs in LNs and that these vessels promote anti-tumour immunity by recruiting naïve T cells into cancerous tissues. This allows T cell priming and reactivation inside solid cancers, thus avoiding the dilution of tumoricidal T cells during redistribution from their LN site of priming *via* the efferent lymphatics and bloodstream (123).

The presence of HEV-containing TLO is highly correlated with active disease and for persistent infections such as *H. pylori*, TLOs in the gastric mucosa disappear when the infection is cleared (125). In the case of solid, vascularized cancers, the presence of TLO in resected solid cancers has been correlated with prolonged patient outcome following resection of the primary cancer in breast cancer (126, 127), melanoma (128, 129), lung (130), and colorectal cancer, although in the latter case the correlation depends on the stage of the disease (131, 132). In separate studies of lung cancer, mature DC containing TLO enriched in CD8^+^ effector memory T cells or expressing a LN-associated chemokine and adhesion molecule gene signature have both been correlated with improved patient outcome (133, 134). Importantly however, the formation of TLO does not always indicate improved cancer patient outcome. In virus-induced hepatic cellular carcinoma, TLOs provide a cytokine-rich niche, which promotes the development and survival of malignant hepatocyte progenitors (11). The density of HEV alone was sufficient to predict patient outcome in breast cancer and melanoma (9, 10), indicating the critical role that HEVs play in orchestrating anticancer immunity. It will be interesting to determine the expression of other markers of HEV differentiation such as MAdCAM-1, GlyCAM-1, GlcNAc6ST-2, and GlcNAc6ST-1 to further understand the precise roles of HEV development and maturation in protective immunity to clinical cancers.

Evidence of ongoing immune responses are seen inside TLOs; activation-induced cytidine deaminase is active in ectopic germinal centers in salivary glands of Sjorgren’s syndrome patients (5) and T cell priming and epitope spreading occur in mouse models of multiple sclerosis and cancer (122, 135). In mice lacking all SLOs, Moyron-Quiroz and colleagues demonstrated that humoral and cellular immune responses develop in TLOs following influenza infection (136). The formation of TLO in clinical conditions may, therefore, reflect a lack of function in draining secondary LNs, which are no longer able to accept incoming antigen or antigen-presenting cells or that SLOs are operating at maximal capacity. The formation of TLOs is thought to exacerbate autoimmune diseases, at least in part, because effector lymphocytes generated within the target organ will not be diluted out during transit from the normal LN site of priming. However, the impact of TLO will depend on the nature of the ongoing immune response to the autoantigen, pathogen, or cancer antigen, which may be protective (CD8^+^ T cells in cancer), inflammatory (Th17 cells in autoimmunity), or tolerance inducing (Foxp3^+^ regulatory T cells), and this may be regulated by the activation status of PNAd-expressing HEVs and additional factors such as chemokines. Further studies are required to understand exactly how ectopic HEVs are formed and their impact on different types of chronic diseases. Such studies may reveal therapeutic targets for intervention in autoimmune diseases and cancers.

### A Unifying Hypothesis of HEV Neogenesis

Extensive studies of SLOs and TLOs have shown that HEVs present as peripheral and/or mucosal addressin expressing blood vessels in which the endothelium is either characteristically cuboidal and filled with transmigrating lymphocytes or flat and, although PNAd-positive, lymphocyte-filled pockets are absent. Can we reconcile these divergent reports of HEV into a single, unifying model of HEV neogenesis in lymphoid and non-lymphoid tissues? Clues for this have come from studies in which cytokines ectopically expressed in pancreatic islets of mice stimulate the development of vascular addressin expressing blood vessels. Expression of LTα-induced PNAd and MAdCAM-1 on pancreatic blood vessels dependent on signaling through TNFR1 but the ECs are flat, do not express the HEV-restricted sulfotransferase (HEC-6ST/GlcNAc6ST-2) and PNAd expression was located to the basolateral EC surface where, in LN, it does not support high levels of lymphocyte recruitment. Structurally distinct, PNAd-expressing blood vessels, similar to HEVs in LN, were only formed when LTα and LTβ were co-expressed in pancreatic islets (137). Interestingly, LT-α drives the development of flat, PNAd-expressing blood vessels in mouse models of cancer which, although structurally similar to immature HEVs in LN, are able to recruit L-selectin-expressing T cells from the bloodstream *via* PNAd^+^ ligands indicating they are not completely immature (124).

LTα and LTαβ activate the classical NF-κB pathway characterized by nuclear translocation of p50–RelA complexes. LTαβ also activates the alternative, non-canonical NF-κB pathway of NF-κB-inducing kinase (NIK)-dependent activation of IκB kinase (IKK)-α and nuclear translocation of p52–RelB complexes (138). Non-canonical NF-κB signaling plays a dominant role in the formation of HEVs in LNs since blockade of LTβR, but not TNFR, leads to loss of several HEV-specific markers such as GlyCAM-1, MAdCAM-1, CCL21, and the HEV-restricted sulfotransferase, HEC-6ST/GlcNAc6ST-2 (58). In addition, PNAd-expressing blood vessels that develop in IKKα(AA) mutant mice where non-canonical NF-κB signaling is defective lack GlyCAM-1 and HEC-6ST (49). Conversely, mice lacking full-length p100 protein, resulting in constitutively active p52, develop PNAd-positive HEVs in the spleen (139). The key event in non-canonical NF-κB activation is signal-induced protein stabilization of NIK that is normally degraded by a ubiquitin ligase complex comprising TRAF2, TRAF3, and cIAP1/2 (140, 141). LTβR ligation sequesters this NIK-targeting destruction complex leading to NIK accumulation. Importantly, signal-induced NIK stability is transient (142), suggesting that continual activation of the LTβR is required to maintain functional NIK expression levels and hence, sustain the activity of the non-canonical NF-κB pathway.

**Figure 2:**
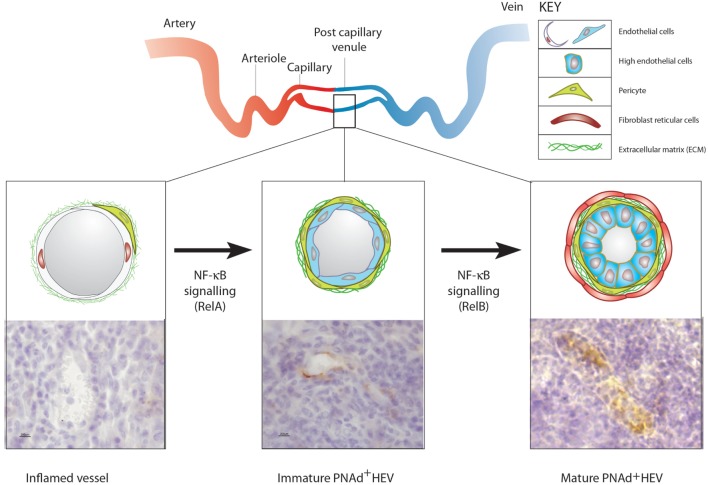
**The development of ectopic high endothelial venules (HEVs)**. Top: diagrammatic representation of blood vasculature in non-lymphoid organs and the location of postcapillary venules. Bottom (left): blood vessels in acutely inflamed tissues do not express vascular addressins but are able to recruit activated lymphoid cells. (Middle) TNFα, LTα, or LTαβ expressing T or NK cells recruited by inflamed blood vessels induce the expression of peripheral node addressin (PNAd) (and/or MAdCAM-1) in blood vessels lined with flat endothelial cells (ECs) by stimulating classical NF-κB (RelA) signaling in ECs. (Right) Sustained contact between LTαβ expressing activated lymphoid cells and PNAd expressing flat EC induces LTβR-dependent non-canonical NF-κB (RelB) signaling in ECs, which allows the full maturation of PNAd-expressing HEV lined with cuboidal ECs filled with transmigrating lymphocytes. It is not known if the recruitment of fibroblast reticular cells into the perivascular sheath surrounding HEV is driven by LTβR signaling in ECs or perivascular cells or both.

It is proposed that the development of HEVs is dissected into at least two distinct stages based on NF-κB signaling in blood vessel ECs (Figure 2). The first stage is driven by LTα-TNFR classical NF-κB signaling and generates MAdCAM-1-expressing HEV lined with flat, PNAd-expressing ECs. The second stage is driven by sustained LTβR non-canonical NF-κB signaling and induces the development of fully mature, PNAd-expressing HEVs lined by HECs and containing lymphocyte-filled pockets. However, these two stages may be not always be clearly delineated because there is overlap between classical and non-canonical NFκB signaling in blood ECs. For example, classical RelA/NF-κB signaling by TNFα, LTα, or LTαβ in EC cultured from human and mouse tissues induces expression of MAdCAM-1 protein and expression of the gene encoding HEC-6ST, which generates the PNAd epitope in LN HEVs (33–35, 143). However, LTβR signaling in isolated human EC has not been reported to induce the expression of PNAd modified glycoproteins (143). It is known that classical NF-κB-dependent signaling in human EC inhibits non-canonical NF-κB signaling (144). The outcome of LTβR signaling in ECs will, therefore, depend on the balance between classical and non-canonical NF-κB signaling. Recent studies have demonstrated that endothelial differentiation is regulated by components of the basal lamina (145). As in other types of postcapillary venule, structural support to the endothelial lining of HEV is provided by the basal lamina, which is known to regulate NF-κB signaling (146). Further studies on isolated blood ECs may identify the stimuli and signaling pathways that stimulate the synthesis of HEV-restricted PNAd-modified glycoproteins thereby controlling HEV neogenesis.

## Therapeutic Strategies to Control HEV Neogenesis and Function

If HEVs in TLO are critical to exacerbation of autoimmune diseases by allowing activation of tissue destroying lymphocytes within target tissues, can the development of HEVs be prevented? Since HEVs in SLOs are important for generating protective immunity to infection any such therapy would need to be targeted to TLO. Intuitively, this will require the identification of markers or signaling pathways in ectopic HEVs that are not shared by HEVs in LN. One candidate is TNFR signaling that is not required for the development or maintenance of HEVs in LN (58) but has been shown to induce PNAd-expressing ectopic HEVs able to recruit naïve T lymphocytes, at least in cancer (124). It is possible that the success of anti-TNF-α or TNFRII-Ig (Etanercept) therapies in rheumatoid arthritis patients may depend, in part, on reversing or blocking blood vessel differentiation toward an HEV-like phenotype. The formation of HEVs in cancerous tissues in the absence of TLO correlates with reduced tumor progression in experimental animals; HEV neogenesis may, therefore, be a possible therapy to control cancer growth but it is not clear how this would be achieved. Clinical and experimental data indicate that tumor blood vessels are poor at recruiting cytotoxic, effector T lymphocytes and present an immune checkpoint that limits effective immunotherapy (147). Several different strategies are being considered to achieve this including targeted delivery of TNF-α to tumor blood vessels, which may induce the development of PNAd-expressing blood vessels although this was not determined (148). Approaches to induce HEV-containing TLO formation in cancers are also being considered but it is worth bearing in mind that TLOs, like SLOs, could be sites of tolerance induction and, therefore, may limit effective antitumor immunity. The full impact of HEVs and/or TLO may only be revealed when highly immunosuppressive cells such as Foxp3^+^ Tregs or myeloid-derived suppressor cells are depleted allowing effector T cells to exit TLO, infiltrate, and kill cancerous tissues (114, 149, 150). It will be important to determine which immune cells are recruited by cancer-associated HEVs to dissect their impact on cancer immunity. Further studies are required to determine the extent and role of HEV development in clinical cancers.

## Author Contributions

The author confirms being the sole contributor of this work and approved it for publication.

## Conflict of Interest Statement

The author declares that the research was conducted in the absence of any commercial or financial relationships that could be construed as a potential conflict of interest.

## References

[1] Masopust D Schenkel JM. The integration of T cell migration, differentiation and function. Nat Rev Immunol (2013) 13(5):309–20. 10.1038/nri3442 23598650

[2] Fletcher AL Malhotra D Turley SJ. Lymph node stroma broaden the peripheral tolerance paradigm. Trends Immunol (2011) 32(1):12–8. 10.1016/j.it.2010.11.002 21147035 PMC3163075

[3] Girard JP Moussion C Forster R. HEVs, lymphatics and homeostatic immune cell trafficking in lymph nodes. Nat Rev Immunol (2012) 12(11):762–73. 10.1038/nri3298 23018291

[4] Drayton DL Liao S Mounzer RH Ruddle NH. Lymphoid organ development: from ontogeny to neogenesis. Nat Immunol (2006) 7(4):344–53. 10.1038/ni1330 16550197

[5] Bombardieri M Barone F Humby F Kelly S McGurk M Morgan P Activation-induced cytidine deaminase expression in follicular dendritic cell networks and interfollicular large B cells supports functionality of ectopic lymphoid neogenesis in autoimmune sialoadenitis and MALT lymphoma in Sjogren’s syndrome. J Immunol (2007) 179(7):4929–38. 10.4049/jimmunol.179.7.4929 17878393

[6] Astorri E Bombardieri M Gabba S Peakman M Pozzilli P Pitzalis C. Evolution of ectopic lymphoid neogenesis and in situ autoantibody production in autoimmune nonobese diabetic mice: cellular and molecular characterization of tertiary lymphoid structures in pancreatic islets. J Immunol (2010) 185(6):3359–68. 10.4049/jimmunol.1001836 20713891

[7] Lee Y Chin RK Christiansen P Sun Y Tumanov AV Wang J Recruitment and activation of naive T cells in the islets by lymphotoxin beta receptor-dependent tertiary lymphoid structure. Immunity (2006) 25(3):499–509. 10.1016/j.immuni.2006.06.016 16934497

[8] Sautes-Fridman C Lawand M Giraldo NA Kaplon H Germain C Fridman WH Tertiary lymphoid structures in cancers: prognostic value, regulation, and manipulation for therapeutic intervention. Front Immunol (2016) 7:407. 10.3389/fimmu.2016.00407 27752258 PMC5046074

[9] Martinet L Garrido I Filleron T Le Guellec S Bellard E Fournie JJ Human solid tumors contain high endothelial venules: association with T- and B-lymphocyte infiltration and favorable prognosis in breast cancer. Cancer Res (2011) 71(17):5678–87. 10.1158/0008-5472.CAN-11-0431 21846823

[10] Martinet L Le Guellec S Filleron T Lamant L Meyer N Rochaix P High endothelial venules (HEVs) in human melanoma lesions: major gateways for tumor-infiltrating lymphocytes. Oncoimmunology (2012) 1(6):829–39. 10.4161/onci.20492 23162750 PMC3489738

[11] Finkin S Yuan D Stein I Taniguchi K Weber A Unger K Ectopic lymphoid structures function as microniches for tumor progenitor cells in hepatocellular carcinoma. Nat Immunol (2015) 16(12):1235–44. 10.1038/ni.3290 26502405 PMC4653079

[12] Wotherspoon AC Doglioni C Diss TC Pan L Moschini A de Boni M Regression of primary low-grade B-cell gastric lymphoma of mucosa-associated lymphoid tissue type after eradication of *Helicobacter pylori*. Lancet (1993) 342(8871):575–7. 10.1016/0140-6736(93)91409-F 8102719

[13] Kobayashi M Lee H Schaffer L Gilmartin TJ Head SR Takaishi S A distinctive set of genes is upregulated during the inflammation-carcinoma sequence in mouse stomach infected b*y Helicobacter felis*. J Histochem Cytochem (2007) 55(3):263–74. 10.1369/jhc.6A7097.2006 17101721

[14] van de Pavert SA Mebius RE. New insights into the development of lymphoid tissues. Nat Rev Immunol (2010) 10(9):664–74. 10.1038/nri2832 20706277

[15] Randall TD Carragher DM Rangel-Moreno J. Development of secondary lymphoid organs. Annu Rev Immunol (2008) 26:627–50. 10.1146/annurev.immunol.26.021607.090257 18370924 PMC2590644

[16] Blum KS Pabst R. Keystones in lymph node development. J Anat (2006) 209(5):585–95. 10.1111/j.1469-7580.2006.00650.x 17062017 PMC2100342

[17] Peduto L Dulauroy S Lochner M Spath GF Morales MA Cumano A Inflammation recapitulates the ontogeny of lymphoid stromal cells. J Immunol (2009) 182(9):5789–99. 10.4049/jimmunol.0803974 19380827

[18] Hendricks HR Eestermans IL. Disappearance and reappearance of high endothelial venules and immigrating lymphocytes in lymph nodes deprived of afferent lymphatic vessels: a possible regulatory role of macrophages in lymphocyte migration. Eur J Immunol (1983) 13:663–9. 10.1002/eji.1830130811 6884423

[19] Mebius RE Streeter PR Breve J Duijvestijn AM Kraal G. The influence of afferent lymphatic vessel interruption on vascular addressin expression. J Cell Biol (1991) 115(1):85–95. 10.1083/jcb.115.1.85 1918141 PMC2289917

[20] Lacorre DA Baekkevold ES Garrido I Brandtzaeg P Haraldsen G Amalric F Plasticity of endothelial cells: rapid dedifferentiation of freshly isolated high endothelial venule endothelial cells outside the lymphoid tissue microenvironment. Blood (2004) 103(11):4164–72. 10.1182/blood-2003-10-3537 14976058

[21] Mebius RE Streeter PR Michie S Butcher EC Weissman IL A developmental switch in lymphocyte homing receptor and endothelial vascular addressin expression regulates lymphocyte homing and permits CD4+ CD3- cells to colonize lymph nodes. Proc Natl Acad Sci U S A (1996) 93(20):11019–24. 10.1073/pnas.93.20.11019 8855301 PMC38276

[22] Mebius RE. Organogenesis of lymphoid tissues. Nat Rev Immunol (2003) 3(4):292–303. 10.1038/nri1054 12669020

[23] Zhang Z Li J Zheng W Zhao G Zhang H Wang X Peripheral lymphoid volume expansion and maintenance are controlled by gut microbiota via RALDH+ dendritic cells. Immunity (2016) 44(2):330–42. 10.1016/j.immuni.2016.01.004 26885858 PMC4757854

[24] Moussion C Girard JP. Dendritic cells control lymphocyte entry to lymph nodes through high endothelial venules. Nature (2011) 479(7374):542–6. 10.1038/nature10540 22080953

[25] Wigle JT Oliver G. Prox1 function is required for the development of the murine lymphatic system. Cell (1999) 98(6):769–78. 10.1016/S0092-8674(00)81511-1 10499794

[26] Mebius RE Rennert P Weissman IL. Developing lymph nodes collect CD4+CD3- LTbeta+ cells that can differentiate to APC, NK cells, and follicular cells but not T or B cells. Immunity (1997) 7(4):493–504. 10.1016/S1074-7613(00)80371-4 9354470

[27] Veiga-Fernandes H Coles MC Foster KE Patel A Williams A Natarajan D Tyrosine kinase receptor RET is a key regulator of Peyer’s patch organogenesis. Nature (2007) 446(7135):547–51. 10.1038/nature05597 17322904

[28] Hashi H Yoshida H Honda K Fraser S Kubo H Awane M Compartmentalization of Peyer’s patch anlagen before lymphocyte entry. J Immunol (2001) 166(6):3702–9. 10.4049/jimmunol.166.6.3702 11238610

[29] Gretz JE Norbury CC Anderson AO Proudfoot AE Shaw S. Lymph-borne chemokines and other low molecular weight molecules reach high endothelial venules via specialized conduits while a functional barrier limits access to the lymphocyte microenvironments in lymph node cortex. J Exp Med (2000) 192(10):1425–40. 10.1084/jem.192.10.1425 11085745 PMC2193184

[30] Pruenster M Mudde L Bombosi P Dimitrova S Zsak M Middleton J The Duffy antigen receptor for chemokines transports chemokines and supports their promigratory activity. Nat Immunol (2009) 10(1):101–8. 10.1038/ni.1675 19060902 PMC3205989

[31] Palframan RT Jung S Cheng CY Weninger W Luo Y Dorf M Inflammatory chemokine transport and presentation in HEV: a remote control mechanism for monocyte recruitment to lymph nodes in inflamed tissues. J Exp Med (2001) 194(9):1361–73. 10.1084/jem.194.9.1361 11696600 PMC2195988

[32] Baekkevold ES Yamanaka T Palframan RT Carlsen HS Reinholt FP von Andrian UH The CCR7 ligand ELC (CCL19) is transcytosed in high endothelial venules and mediates T cell recruitment. J Exp Med (2001) 193(9):1105–12. 10.1084/jem.193.9.1105 11342595 PMC2193428

[33] Sikorski EE Hallmann R Berg EL Butcher EC. The Peyer’s patch high endothelial receptor for lymphocytes, the mucosal vascular addressin, is induced on a murine endothelial cell line by tumor necrosis factor-alpha and IL-1. J Immunol (1993) 151(10):5239–50. 7693807

[34] Takeuchi M Baichwal VR. Induction of the gene encoding mucosal vascular addressin cell adhesion molecule 1 by tumor necrosis factor alpha is mediated by NF-kappa B proteins. Proc Natl Acad Sci U S A (1995) 92(8):3561–5. 10.1073/pnas.92.8.3561 7724598 PMC42207

[35] Cuff CA Schwartz J Bergman CM Russell KS Bender JR Ruddle NH. Lymphotoxin alpha3 induces chemokines and adhesion molecules: insight into the role of LT alpha in inflammation and lymphoid organ development. J Immunol (1998) 161(12):6853–60. 9862717

[36] Pabst O Forster R Lipp M Engel H Arnold HH. NKX2.3 is required for MAdCAM-1 expression and homing of lymphocytes in spleen and mucosa-associated lymphoid tissue. EMBO J (2000) 19(9):2015–23. 10.1093/emboj/19.9.2015 10790368 PMC305695

[37] Schippers A Leuker C Pabst O Kochut A Prochnow B Gruber AD Mucosal addressin cell-adhesion molecule-1 controls plasma-cell migration and function in the small intestine of mice. Gastroenterology (2009) 137(3):924–33. 10.1053/j.gastro.2009.05.039 19450594

[38] Gurtner GC Davis V Li H McCoy MJ Sharpe A Cybulsky MI. Targeted disruption of the murine VCAM1 gene: essential role of VCAM-1 in chorioallantoic fusion and placentation. Genes Dev (1995) 9(1):1–14. 10.1101/gad.9.1.1 7530222

[39] Xu H Gonzalo JA St Pierre Y Williams IR Kupper TS Cotran RS Leukocytosis and resistance to septic shock in intercellular adhesion molecule 1-deficient mice. J Exp Med (1994) 180(1):95–109. 10.1084/jem.180.1.95 7911822 PMC2191562

[40] De Togni P Goellner J Ruddle NH Streeter PR Fick A Mariathasan S Abnormal development of peripheral lymphoid organs in mice deficient in lymphotoxin. Science (1994) 264(5159):703–7. 10.1126/science.8171322 8171322

[41] Rennert PD James D Mackay F Browning JL Hochman PS. Lymph node genesis is induced by signaling through the lymphotoxin beta receptor. Immunity (1998) 9(1):71–9. 10.1016/S1074-7613(00)80589-0 9697837

[42] Rennert PD Browning JL Mebius R Mackay F Hochman PS. Surface lymphotoxin alpha/beta complex is required for the development of peripheral lymphoid organs. J Exp Med (1996) 184(5):1999–2006. 10.1084/jem.184.5.1999 8920886 PMC2192901

[43] Lai L Bohnsack BL Niederreither K Hirschi KK. Retinoic acid regulates endothelial cell proliferation during vasculogenesis. Development (2003) 130(26):6465–74. 10.1242/dev.00887 14627725

[44] Rennert PD Browning JL Hochman PS. Selective disruption of lymphotoxin ligands reveals a novel set of mucosal lymph nodes and unique effects on lymph node cellular organization. Int Immunol (1997) 9(11):1627–39. 10.1093/intimm/9.11.1627 9418124

[45] May MJ Ghosh S Signal transduction through NF-kappa B. Immunol Today (1998) 19(2):80–8. 10.1016/S0167-5699(97)01197-3 9509763

[46] Alcamo E Hacohen N Schulte LC Rennert PD Hynes RO Baltimore D. Requirement for the NF-kappaB family member RelA in the development of secondary lymphoid organs. J Exp Med (2002) 195(2):233–44. 10.1084/jem.20011885 11805150 PMC2193608

[47] Weih F Carrasco D Durham SK Barton DS Rizzo CA Ryseck RP Multiorgan inflammation and hematopoietic abnormalities in mice with a targeted disruption of RelB, a member of the NF-kappa B/Rel family. Cell (1995) 80(2):331–40. 10.1016/0092-8674(95)90416-6 7834753

[48] Carragher D Johal R Button A White A Eliopoulos A Jenkinson E A stroma-derived defect in NF-kappaB2-/- mice causes impaired lymph node development and lymphocyte recruitment. J Immunol (2004) 173(4):2271–9. 10.4049/jimmunol.173.4.2271 15294939

[49] Drayton DL Bonizzi G Ying X Liao S Karin M Ruddle NH I kappa B kinase complex alpha kinase activity controls chemokine and high endothelial venule gene expression in lymph nodes and nasal-associated lymphoid tissue. J Immunol (2004) 173(10):6161–8. 10.4049/jimmunol.173.10.6161 15528353

[50] Onder L Danuser R Scandella E Firner S Chai Q Hehlgans T Endothelial cell-specific lymphotoxin-beta receptor signaling is critical for lymph node and high endothelial venule formation. J Exp Med (2013) 210(3):465–73. 10.1084/jem.20121462 23420877 PMC3600902

[51] Rosen SD. Ligands for L-selectin: homing, inflammation, and beyond. Annu Rev Immunol (2004) 22:129–56. 10.1146/annurev.immunol.21.090501.080131 15032576

[52] Mitoma J Bao X Petryanik B Schaerli P Gauguet JM Yu SY Critical functions of N-glycans in L-selectin-mediated lymphocyte homing and recruitment. Nat Immunol (2007) 8(4):409–18. 10.1038/ni1442 17334369

[53] Streeter PR Rouse BT Butcher EC. Immunohistologic and functional characterization of a vascular addressin involved in lymphocyte homing into peripheral lymph nodes. J Cell Biol (1988) 107(5):1853–62. 10.1083/jcb.107.5.1853 2460470 PMC2115336

[54] Streeter PR Berg EL Rouse BT Bargatze RF Butcher EC. A tissue-specific endothelial cell molecule involved in lymphocyte homing. Nature (1988) 331(6151):41–6. 10.1038/331041a0 3340147

[55] Kellermayer Z Labadi A Czompoly T Arnold HH Balogh P. Absence of Nkx2-3 homeodomain transcription factor induces the formation of LYVE-1-positive endothelial cysts without lymphatic commitment in the spleen. J Histochem Cytochem (2011) 59(7):690–700. 10.1369/0022155411410061 21705651 PMC3201159

[56] Anderson AO Shaw S. T cell adhesion to endothelium: the FRC conduit system and other anatomic and molecular features which facilitate the adhesion cascade in lymph node. Semin Immunol (1993) 5(4):271–82. 10.1006/smim.1993.1031 8219105

[57] Swarte VV Joziasse DH Van den Eijnden DH Petryniak B Lowe JB Kraal G Regulation of fucosyltransferase-VII expression in peripheral lymph node high endothelial venules. Eur J Immunol (1998) 28(10):3040–7. 10.1002/(SICI)1521-4141(199810)28:10<3040::AID-IMMU3040>3.0.CO;2-5 9808172

[58] Browning JL Allaire N Ngam-Ek A Notidis E Hunt J Perrin S Lymphotoxin-beta receptor signaling is required for the homeostatic control of HEV differentiation and function. Immunity (2005) 23(5):539–50. 10.1016/j.immuni.2005.10.002 16286021

[59] Lindquist RL Shakhar G Dudziak D Wardemann H Eisenreich T Dustin ML Visualizing dendritic cell networks in vivo. Nat Immunol (2004) 5(12):1243–50. 10.1038/ni1139 15543150

[60] Kissenpfennig A Henri S Dubois B Laplace-Builhe C Perrin P Romani N Dynamics and function of Langerhans cells in vivo: dermal dendritic cells colonize lymph node areas distinct from slower migrating Langerhans cells. Immunity (2005) 22(5):643–54. 10.1016/j.immuni.2005.04.004 15894281

[61] Bajenoff M Granjeaud S Guerder S. The strategy of T cell antigen-presenting cell encounter in antigen-draining lymph nodes revealed by imaging of initial T cell activation. J Exp Med (2003) 198(5):715–24. 10.1084/jem.20030167 12953093 PMC2194192

[62] Randolph GJ Ochando J Partida-Sanchez S. Migration of dendritic cell subsets and their precursors. Annu Rev Immunol (2008) 26:293–316. 10.1146/annurev.immunol.26.021607.090254 18045026

[63] Choi JH Do Y Cheong C Koh H Boscardin SB Oh YS Identification of antigen-presenting dendritic cells in mouse aorta and cardiac valves. J Exp Med (2009) 206(3):497–505. 10.1084/jem.20082129 19221394 PMC2699134

[64] Wendland M Willenzon S Kocks J Davalos-Misslitz AC Hammerschmidt SI Schumann K Lymph node T cell homeostasis relies on steady state homing of dendritic cells. Immunity (2011) 35(6):945–57. 10.1016/j.immuni.2011.10.017 22195748

[65] Chyou S Ekland EH Carpenter AC Tzeng TC Tian S Michaud M Fibroblast-type reticular stromal cells regulate the lymph node vasculature. J Immunol (2008) 181(6):3887–96. 10.4049/jimmunol.181.6.3887 18768843 PMC2562332

[66] Webster B Ekland EH Agle LM Chyou S Ruggieri R Lu TT. Regulation of lymph node vascular growth by dendritic cells. J Exp Med (2006) 203(8):1903–13. 10.1084/jem.20052272 16831898 PMC2118366

[67] Ley K Laudanna C Cybulsky MI Nourshargh S. Getting to the site of inflammation: the leukocyte adhesion cascade updated. Nat Rev Immunol (2007) 7(9):678–89. 10.1038/nri2156 17717539

[68] Steiniger B Timphus EM Barth PJ. The splenic marginal zone in humans and rodents: an enigmatic compartment and its inhabitants. Histochem Cell Biol (2006) 126(6):641–8. 10.1007/s00418-006-0210-5 16816939

[69] Lee M Kiefel H LaJevic MD Macauley MS Kawashima H O’Hara E Transcriptional programs of lymphoid tissue capillary and high endothelium reveal control mechanisms for lymphocyte homing. Nat Immunol (2014) 15(10):982–95. 10.1038/ni.2983 25173345 PMC4222088

[70] Warnock RA Askari S Butcher EC von Andrian UH. Molecular mechanisms of lymphocyte homing to peripheral lymph nodes. J Exp Med (1998) 187(2):205–16. 10.1084/jem.187.2.205 9432978 PMC2212097

[71] M’Rini C Cheng G Schweitzer C Cavanagh LL Palframan RT Mempel TR A novel endothelial L-selectin ligand activity in lymph node medulla that is regulated by alpha(1,3)-fucosyltransferase-IV.[see comment]. J Exp Med (2003) 198(9):1301–12. 10.1084/jem.20030182 14597733 PMC2194247

[72] Picker LJ Butcher EC. Physiological and molecular mechanisms of lymphocyte homing. Annu Rev Immunol (1992) 10:561–91. 10.1146/annurev.iy.10.040192.003021 1590996

[73] Gauguet JM Bonaiso R von Andrian UH High endothelial venules. In: Aird WC, editor. Endothelial BioMedcine. New York, NY: Cambridge University Press (2007). p. 1568–88.

[74] Ager A Coles MC Stein JV Development of lymph node circulation and homing mechanisms. In: Balogh P, editor. Developmental Biology of Lymphoid Organs. Berlin: Springer-Verlag (2010). p. 75–94.

[75] Michie SA Streeter PR Bolt PA Butcher EC Picker LJ. The human peripheral lymph node vascular addressin. An inducible endothelial antigen involved in lymphocyte homing. Am J Pathol (1993) 143(6):1688–98. 8256856 PMC1887255

[76] Abitorabi MA Mackay CR Jerome EH Osorio O Butcher EC Erle DJ. Differential expression of homing molecules on recirculating lymphocytes from sheep gut, peripheral, and lung lymph. J Immunol (1996) 156(9):3111–7. 8617931

[77] Briskin M Winsor-Hines D Shyjan A Cochran N Bloom S Wilson J Human mucosal addressin cell adhesion molecule-1 is preferentially expressed in intestinal tract and associated lymphoid tissue. Am J Pathol (1997) 151(1):97–110. 9212736 PMC1857942

[78] Pullen N Molloy E Carter D Syntin P Clemo F Finco-Kent D Pharmacological characterization of PF-00547659, an anti-human MAdCAM monoclonal antibody. Br J Pharmacol (2009) 157(2):281–93. 10.1111/j.1476-5381.2009.00137.x 19366349 PMC2697799

[79] Salmi M Alanen K Grenman S Briskin M Butcher EC Jalkanen S. Immune cell trafficking in uterus and early life is dominated by the mucosal addressin MAdCAM-1 in humans. Gastroenterology (2001) 121(4):853–64. 10.1053/gast.2001.27968 11606499

[80] Arbones ML Ord DC Ley K Ratech H Maynard-Curry C Otten G Lymphocyte homing and leukocyte rolling and migration are impaired in L-selectin-deficient mice. Immunity (1994) 1(4):247–60. 10.1016/1074-7613(94)90076-0 7534203

[81] Fossum S Smith ME Ford WL. The migration of lymphocytes across specialized vascular endothelium VII. The migration of T and B lymphocytes from the blood of the athymic, nude rat. Scand J Immunol (1983) 17(6):539–50. 10.1111/j.1365-3083.1983.tb00822.x 6603012

[82] Bajenoff M Egen JG Koo LY Laugier JP Brau F Glaichenhaus N Stromal cell networks regulate lymphocyte entry, migration, and territoriality in lymph nodes. Immunity (2006) 25(6):989–1001. 10.1016/j.immuni.2006.10.011 17112751 PMC2692293

[83] Boscacci RT Pfeiffer F Gollmer K Sevilla AI Martin AM Soriano SF Comprehensive analysis of lymph node stroma-expressed Ig superfamily members reveals redundant and nonredundant roles for ICAM-1, ICAM-2, and VCAM-1 in lymphocyte homing. Blood (2010) 116(6):915–25. 10.1182/blood-2009-11-254334 20395417 PMC3324225

[84] Schoefl GI. The migration of lymphocytes across the vascular endothelium in lymphoid tissue. A reexamination. J Exp Med (1972) 136(3):568–88. 10.1084/jem.136.3.568 5050723 PMC2139256

[85] Engelhardt B Wolburg H. Mini-review: transendothelial migration of leukocytes: through the front door or around the side of the house? Eur J Immunol (2004) 34(11):2955–63. 10.1002/eji.200425327 15376193

[86] Mionnet C Sanos SL Mondor I Jorquera A Laugier JP Germain RN High endothelial venules as traffic control points maintaining lymphocyte population homeostasis in lymph nodes. Blood (2011) 118(23):6115–22. 10.1182/blood-2011-07-367409 21937697 PMC3234668

[87] Faveeuw C Preece G Ager A. Transendothelial migration of lymphocytes across high endothelial venules into lymph nodes is affected by metalloproteinases. Blood (2001) 98(3):688–95. 10.1182/blood.V98.3.688 11468168

[88] Sainte-Marie G Peng FS. Dilatation of high endothelial venules in compartments of rat lymph nodes with abundant cortical mast cells. J Anat (1991) 174:163–70. 2032932 PMC1256052

[89] Belisle C Sainte-Marie G. The narrowing of high endothelial venules of the rat lymph node. Anat Rec (1985) 211(2):184–91. 10.1002/ar.1092110210 3883847

[90] Campbell FR. Intercellular contacts of lymphocytes during migration across high-endothelial venules of lymph nodes. An electron microscopic study. Anat Rec (1983) 207(4):643–52. 10.1002/ar.1092070413 6670759

[91] Barreiro O Yanez-Mo M Serrador JM Montoya MC Vicente-Manzanares M Tejedor R Dynamic interaction of VCAM-1 and ICAM-1 with moesin and ezrin in a novel endothelial docking structure for adherent leukocytes. J Cell Biol (2002) 157(7):1233–45. 10.1083/jcb.200112126 12082081 PMC2173557

[92] Carman CV Springer TA. A transmigratory cup in leukocyte diapedesis both through individual vascular endothelial cells and between them. J Cell Biol (2004) 167(2):377–88. 10.1083/jcb.200404129 15504916 PMC2172560

[93] Pfeiffer F Kumar V Butz S Vestweber D Imhof BA Stein JV Distinct molecular composition of blood and lymphatic vascular endothelial cell junctions establishes specific functional barriers within the peripheral lymph node. Eur J Immunol (2008) 38(8):2142–55. 10.1002/eji.200838140 18629939

[94] Ager A May MJ. Understanding high endothelial venules: lessons for cancer immunology. Oncoimmunology (2015) 4(6):e1008791. 10.1080/2162402X.2015.1008791 26155419 PMC4485764

[95] Halin C Scimone ML Bonasio R Gauguet JM Mempel TR Quackenbush E The S1P-analog FTY720 differentially modulates T-cell homing via HEV: T-cell-expressed S1P1 amplifies integrin activation in peripheral lymph nodes but not in Peyer patches. Blood (2005) 106(4):1314–22. 10.1182/blood-2004-09-3687 15870184 PMC1895188

[96] Hourihan H Allen TD Ager A. Lymphocyte migration across high endothelium is associated with increases in alpha 4 beta 1 integrin (VLA-4) affinity. J Cell Sci (1993) 104(Pt 4):1049–59. 8314890 10.1242/jcs.104.4.1049

[97] Klinger A Gebert A Bieber K Kalies K Ager A Bell EB Cyclical expression of L-selectin (CD62L) by recirculating T cells. Int Immunol (2009) 21(4):443–55. 10.1093/intimm/dxp012 19240088

[98] Galkina E Tanousis K Preece G Tolaini M Kioussis D Florey O L-selectin shedding does not regulate constitutive T cell trafficking but controls the migration pathways of antigen-activated T lymphocytes. J Exp Med (2003) 198(9):1323–35. 10.1084/jem.20030485 14597735 PMC2194256

[99] Rzeniewicz K Newe A Rey Gallardo A Davies J Holt MR Patel A L-selectin shedding is activated specifically within transmigrating pseudopods of monocytes to regulate cell polarity in vitro. Proc Natl Acad Sci U S A (2015) 112(12):E1461–70. 10.1073/pnas.1417100112 25775539 PMC4378423

[100] Hickey MJ Forster M Mitchell D Kaur J De Caigny C Kubes P. L-selectin facilitates emigration and extravascular locomotion of leukocytes during acute inflammatory responses in vivo. J Immunol (2000) 165(12):7164–70. 10.4049/jimmunol.165.12.7164 11120848

[101] Bai Z Cai L Umemoto E Takeda A Tohya K Komai Y Constitutive lymphocyte transmigration across the basal lamina of high endothelial venules is regulated by the autotaxin/lysophosphatidic acid axis. J Immunol (2013) 190(5):2036–48. 10.4049/jimmunol.1202025 23365076

[102] Kanda H Newton R Klein R Morita Y Gunn MD Rosen SD. Autotaxin, an ectoenzyme that produces lysophosphatidic acid, promotes the entry of lymphocytes into secondary lymphoid organs. Nat Immunol (2008) 9(4):415–23. 10.1038/ni1573 18327261 PMC2783613

[103] Soderberg KA Payne GW Sato A Medzhitov R Segal SS Iwasaki A. Innate control of adaptive immunity via remodeling of lymph node feed arteriole. Proc Natl Acad Sci U S A (2005) 102(45):16315–20. 10.1073/pnas.0506190102 16260739 PMC1283434

[104] Hay JB Hobbs BB. The flow of blood to lymph nodes and its relation to lymphocyte traffic and the immune response. J Exp Med (1977) 145(1):31–44. 10.1084/jem.145.1.31 830789 PMC2180596

[105] Kumar V Scandella E Danuser R Onder L Nitschke M Fukui Y Global lymphoid tissue remodeling during a viral infection is orchestrated by a B cell-lymphotoxin-dependent pathway. Blood (2010) 115(23):4725–33. 10.1182/blood-2009-10-250118 20185585

[106] Chyou S Benahmed F Chen J Kumar V Tian S Lipp M Coordinated regulation of lymph node vascular-stromal growth first by CD11c+ cells and then by T and B cells. J Immunol (2011) 187(11):5558–67. 10.4049/jimmunol.1101724 22031764 PMC3221869

[107] Liao S Ruddle NH. Synchrony of high endothelial venules and lymphatic vessels revealed by immunization. J Immunol (2006) 177(5):3369–79. 10.4049/jimmunol.177.5.3369 16920978

[108] Sawa S Cherrier M Lochner M Satoh-Takayama N Fehling HJ Langa F Lineage relationship analysis of RORgammat+ innate lymphoid cells. Science (2010) 330(6004):665–9. 10.1126/science.1194597 20929731

[109] Scandella E Bolinger B Lattmann E Miller S Favre S Littman DR Restoration of lymphoid organ integrity through the interaction of lymphoid tissue-inducer cells with stroma of the T cell zone. Nat Immunol (2008) 9(6):667–75. 10.1038/ni.1605 18425132

[110] Mueller SN Hosiawa-Meagher KA Konieczny BT Sullivan BM Bachmann MF Locksley RM Regulation of homeostatic chemokine expression and cell trafficking during immune responses. Science (2007) 317(5838):670–4. 10.1126/science.1144830 17673664

[111] Tzeng TC Chyou S Tian S Webster B Carpenter AC Guaiquil VH CD11c(hi) dendritic cells regulate the re-establishment of vascular quiescence and stabilization after immune stimulation of lymph nodes. J Immunol (2010) 184(8):4247–57. 10.4049/jimmunol.0902914 20231692 PMC5815168

[112] Pitzalis C Jones GW Bombardieri M Jones SA. Ectopic lymphoid-like structures in infection, cancer and autoimmunity. Nat Rev Immunol (2014) 14(7):447–62. 10.1038/nri3700 24948366

[113] Cipponi A Mercier M Seremet T Baurain JF Theate I van den Oord J Neogenesis of lymphoid structures and antibody responses occur in human melanoma metastases. Cancer Res (2012) 72(16):3997–4007. 10.1158/0008-5472.CAN-12-1377 22850419

[114] Hindley JP Jones E Smart K Bridgeman H Lauder SN Ondondo B T-cell trafficking facilitated by high endothelial venules is required for tumor control after regulatory T-cell depletion. Cancer Res (2012) 72(21):5473–82. 10.1158/0008-5472.CAN-12-1912 22962270 PMC3491872

[115] Luther SA Lopez T Bai W Hanahan D Cyster JG. BLC expression in pancreatic islets causes B cell recruitment and lymphotoxin-dependent lymphoid neogenesis. Immunity (2000) 12(5):471–81. 10.1016/S1074-7613(00)80199-5 10843380

[116] Luther SA Bidgol A Hargreaves DC Schmidt A Xu Y Paniyadi J Differing activities of homeostatic chemokines CCL19, CCL21, and CXCL12 in lymphocyte and dendritic cell recruitment and lymphoid neogenesis. J Immunol (2002) 169(1):424–33. 10.4049/jimmunol.169.1.424 12077273

[117] Martin AP Coronel EC Sano G Chen SC Vassileva G Canasto-Chibuque C A novel model for lymphocytic infiltration of the thyroid gland generated by transgenic expression of the CC chemokine CCL21. J Immunol (2004) 173(8):4791–8. 10.4049/jimmunol.173.8.4791 15470018

[118] Drayton DL Ying X Lee J Lesslauer W Ruddle NH. Ectopic LT alpha beta directs lymphoid organ neogenesis with concomitant expression of peripheral node addressin and a HEV-restricted sulfotransferase. J Exp Med (2003) 197(9):1153–63. 10.1084/jem.20021761 12732657 PMC2193975

[119] Barone F Nayar S Campos J Cloake T Withers DR Toellner KM IL-22 regulates lymphoid chemokine production and assembly of tertiary lymphoid organs. Proc Natl Acad Sci U S A (2015) 112(35):11024–9. 10.1073/pnas.1503315112 26286991 PMC4568258

[120] Sacca R Cuff CA Lesslauer W Ruddle NH. Differential activities of secreted lymphotoxin-alpha3 and membrane lymphotoxin-alpha1beta2 in lymphotoxin-induced inflammation: critical role of TNF receptor 1 signaling. J Immunol (1998) 160(1):485–91. 9552007

[121] Marinkovic T Garin A Yokota Y Fu YX Ruddle NH Furtado GC Interaction of mature CD3+CD4+ T cells with dendritic cells triggers the development of tertiary lymphoid structures in the thyroid. J Clin Invest (2006) 116(10):2622–32. 10.1172/JCI28993 16998590 PMC1570377

[122] Schrama D thor Straten P Fischer WH McLellan AD Brocker EB Reisfeld RA Targeting of lymphotoxin-alpha to the tumor elicits an efficient immune response associated with induction of peripheral lymphoid-like tissue. Immunity (2001) 14(2):111–21. 10.1016/S1074-7613(01)00094-2 11239444

[123] Yu P Lee Y Liu W Chin RK Wang J Wang Y Priming of naive T cells inside tumors leads to eradication of established tumors. Nat Immunol (2004) 5(2):141–9. 10.1038/ni1029 14704792

[124] Peske JD Thompson ED Gemta L Baylis RA Fu YX Engelhard VH. Effector lymphocyte-induced lymph node-like vasculature enables naive T-cell entry into tumours and enhanced anti-tumour immunity. Nat Commun (2015) 6:7114. 10.1038/ncomms8114 25968334 PMC4435831

[125] Genta RM Hamner HW. The significance of lymphoid follicles in the interpretation of gastric biopsy specimens. Arch Pathol Lab Med (1994) 118(7):740–3. 7517659

[126] Gu-Trantien C Loi S Garaud S Equeter C Libin M de Wind A CD4(+) follicular helper T cell infiltration predicts breast cancer survival. J Clin Invest (2013) 123(7):2873–92. 10.1172/JCI67428 23778140 PMC3696556

[127] Lee HJ Park IA Song IH Shin SJ Kim JY Yu JH Tertiary lymphoid structures: prognostic significance and relationship with tumour-infiltrating lymphocytes in triple-negative breast cancer. J Clin Pathol (2016) 69(5):422–30. 10.1136/jclinpath-2015-203089 26475777

[128] Ladanyi A Kiss J Somlai B Gilde K Fejos Z Mohos A Density of DC-LAMP(+) mature dendritic cells in combination with activated T lymphocytes infiltrating primary cutaneous melanoma is a strong independent prognostic factor. Cancer Immunol Immunother (2007) 56(9):1459–69. 10.1007/s00262-007-0286-3 17279413 PMC11030123

[129] Messina JL Fenstermacher DA Eschrich S Qu X Berglund AE Lloyd MC 12-Chemokine gene signature identifies lymph node-like structures in melanoma: potential for patient selection for immunotherapy? Sci Rep (2012) 2:765. 10.1038/srep00765 23097687 PMC3479449

[130] Dieu-Nosjean MC Antoine M Danel C Heudes D Wislez M Poulot V Long-term survival for patients with non-small-cell lung cancer with intratumoral lymphoid structures. J Clin Oncol (2008) 26(27):4410–7. 10.1200/JCO.2007.15.0284 18802153

[131] Di Caro G Bergomas F Grizzi F Doni A Bianchi P Malesci A Occurrence of tertiary lymphoid tissue is associated with T-cell infiltration and predicts better prognosis in early-stage colorectal cancers. Clin Cancer Res (2014) 20(8):2147–58. 10.1158/1078-0432.CCR-13-2590 24523438

[132] Bento DC Jones E Junaid S Tull J Williams GT Godkin A High endothelial venules are rare in colorectal cancers but accumulate in extra-tumoral areas with disease progression. Oncoimmunology (2015) 4(3):e974374. 10.4161/2162402X.2014.974374 25949892 PMC4404788

[133] Goc J Germain C Vo-Bourgais TK Lupo A Klein C Knockaert S Dendritic cells in tumor-associated tertiary lymphoid structures signal a Th1 cytotoxic immune contexture and license the positive prognostic value of infiltrating CD8+ T cells. Cancer Res (2014) 74(3):705–15. 10.1158/0008-5472.CAN-13-1342 24366885

[134] de Chaisemartin L Goc J Damotte D Validire P Magdeleinat P Alifano M Characterization of chemokines and adhesion molecules associated with T cell presence in tertiary lymphoid structures in human lung cancer. Cancer Res (2011) 71(20):6391–9. 10.1158/0008-5472.CAN-11-0952 21900403

[135] McMahon EJ Bailey SL Castenada CV Waldner H Miller SD. Epitope spreading initiates in the CNS in two mouse models of multiple sclerosis. Nat Med (2005) 11(3):335–9. 10.1038/nm1202 15735651

[136] Moyron-Quiroz JE Rangel-Moreno J Kusser K Hartson L Sprague F Goodrich S Role of inducible bronchus associated lymphoid tissue (iBALT) in respiratory immunity. Nat Med (2004) 10(9):927–34. 10.1038/nm1091 15311275

[137] Cuff CA Sacca R Ruddle NH. Differential induction of adhesion molecule and chemokine expression by LTalpha3 and LTalphabeta in inflammation elucidates potential mechanisms of mesenteric and peripheral lymph node development. J Immunol (1999) 162(10):5965–72. 10229834

[138] Sun SC The noncanonical NF-kappaB pathway. Immunol Rev (2012) 246(1):125–40. 10.1111/j.1600-065X.2011.01088.x 22435551 PMC3313452

[139] Guo F Weih D Meier E Weih F. Constitutive alternative NF-kappaB signaling promotes marginal zone B-cell development but disrupts the marginal sinus and induces HEV-like structures in the spleen. Blood (2007) 110(7):2381–9. 10.1182/blood-2007-02-075143 17620454

[140] Zarnegar BJ Wang Y Mahoney DJ Dempsey PW Cheung HH He J Noncanonical NF-kappaB activation requires coordinated assembly of a regulatory complex of the adaptors cIAP1, cIAP2, TRAF2 and TRAF3 and the kinase NIK. Nat Immunol (2008) 9(12):1371–8. 10.1038/ni.1676 18997794 PMC2676931

[141] Vallabhapurapu S Matsuzawa A Zhang W Tseng PH Keats JJ Wang H Nonredundant and complementary functions of TRAF2 and TRAF3 in a ubiquitination cascade that activates NIK-dependent alternative NF-kappaB signaling. Nat Immunol (2008) 9(12):1364–70. 10.1038/ni.1678 18997792 PMC2671996

[142] Razani B Zarnegar B Ytterberg AJ Shiba T Dempsey PW Ware CF Negative feedback in noncanonical NF-kappaB signaling modulates NIK stability through IKKalpha-mediated phosphorylation. Sci Signal (2010) 3(123):ra41. 10.1126/scisignal.2000778 20501937 PMC2913610

[143] Pablos JL Santiago B Tsay D Singer MS Palao G Galindo M A HEV-restricted sulfotransferase is expressed in rheumatoid arthritis synovium and is induced by lymphotoxin-alpha/beta and TNF-alpha in cultured endothelial cells. BMC Immunol (2005) 6:6. 10.1186/1471-2172-6-6 15752429 PMC1079838

[144] Madge LA May MJ. Classical NF-kappaB activation negatively regulates noncanonical NF-kappaB-dependent CXCL12 expression. J Biol Chem (2010) 285(49):38069–77. 10.1074/jbc.M110.147207 20923761 PMC2992241

[145] Cooley LS Handsley MM Zhou Z Lafleur MA Pennington CJ Thompson EW Reversible transdifferentiation of blood vascular endothelial cells to a lymphatic-like phenotype in vitro. J Cell Sci (2010) 123(Pt 21):3808–16. 10.1242/jcs.064279 20940254

[146] Klein S de Fougerolles AR Blaikie P Khan L Pepe A Green CD Alpha 5 beta 1 integrin activates an NF-kappa B-dependent program of gene expression important for angiogenesis and inflammation. Mol Cell Biol (2002) 22(16):5912–22. 10.1128/MCB.22.16.5912-5922.2002 12138201 PMC133962

[147] Ager A Watson HA Wehenkel SC Mohammed RN. Homing to solid cancers: a vascular checkpoint in adoptive cell therapy using CAR T-cells. Biochem Soc Trans (2016) 44(2):377–85. 10.1042/BST20150254 27068943 PMC5264496

[148] Calcinotto A Grioni M Jachetti E Curnis F Mondino A Parmiani G Targeting TNF-alpha to neoangiogenic vessels enhances lymphocyte infiltration in tumors and increases the therapeutic potential of immunotherapy. J Immunol (2012) 188(6):2687–94. 10.4049/jimmunol.1101877 22323546

[149] Joshi NS Akama-Garren EH Lu Y Lee DY Chang GP Li A Regulatory T cells in tumor-associated tertiary lymphoid structures suppress anti-tumor T cell responses. Immunity (2015) 43(3):579–90. 10.1016/j.immuni.2015.08.006 26341400 PMC4826619

[150] Lanca T Silva-Santos B. The split nature of tumor-infiltrating leukocytes: implications for cancer surveillance and immunotherapy. Oncoimmunology (2012) 1(5):717–25. 10.4161/onci.20068 22934263 PMC3429575

